# Adiabatic Energetic Annealing via Dual Single-Pixel Detection in an Optical Nonlinear Ising Machine

**DOI:** 10.1021/acsphotonics.4c02496

**Published:** 2025-04-14

**Authors:** Luana Olivieri, Andrew R. Cooper, Luke Peters, Vittorio Cecconi, Alessia Pasquazi, Marco Peccianti, Juan S. Totero Gongora

**Affiliations:** Emergent Photonics Research Centre, Department of Physics, 5156 Loughborough University, LE11 3TU, Loughborough, United Kingdom

**Keywords:** optical computing, Ising machines, spin-glass, computational single-pixel sensing

## Abstract

Photonic Ising machines are leading key advancements in solving large combinatorial problems, leveraging large-scale platforms with parallel computing capabilities. A well-known bottleneck of complex problems is the appearance of multiple minima in the energetic landscape that attract Metropolis-based iterations in suboptimal solutions, thus hindering the performance of standard optical solvers in large systems. By introducing a double single-pixel detection scheme based on intensity and field averages in an optical-based Ising machine, we effectively implement local and nonlocal nonlinear Hamiltonians, representing a complex and simple state, respectively. Transitioning from nonlocal to local nonlinear detection enables to adiabatically morph the energetic landscape, enhancing the success rate of finding the optimal solution compared to standard isothermal approaches.

## Graphical abstract

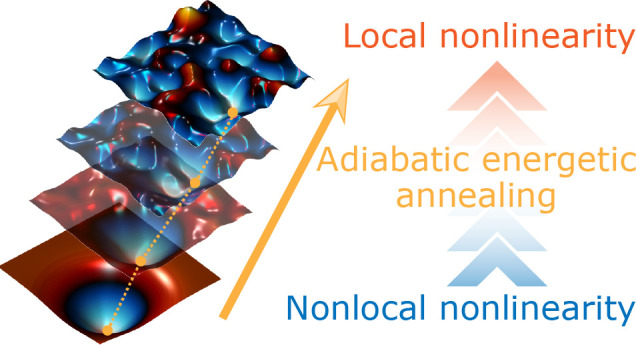

## Introduction

Hard combinatorial problems^1^ require the discovery of optimal solutions, a task that demands computational time scaling exponentially, or in a nondeterministic polynomial time (NP), with the size of the problem.^2^ As many complex problems can be mapped into an Ising Hamiltonian, Ising machines^3–8^ have emerged as new computational hardware bridging the field of statistical mechanics and combinatorial optimization,^2,9^ with impact in many areas such as polymer folding, complex circuit design, and decision-based subjects like finance and social science. Among different optical-based Ising machines that associate a state of light with the spin and encode the coupling problem in the light modulation,^10^ spatial photonic Ising machines^11–14^ are particularly suited to combine extreme parallelization and fast measurements. They encode the spin configuration and the coupling coefficients by spatially modulating a continuous wave (CW) laser light with multiple spatial-light modulators, and the measurement of the intensity, corresponding to the calculation of the Hamiltonian, takes place in a camera or detector at the center of the Fourier plane. While the system has already proven optimization of large-scale systems of the order of thousands of variables,^15^ the protocol for encoding the coupling coefficients limits the implementation of dense graphs, scaling as $\sqrt{M}$ , where *M* is the number of pixels on the spatial light modulator. Recent advances have tackled this problem for sparse graphs,^16^ by either employing wavelength-division multiplexing^17^ or multiple detections via focal plane division,^18^ which are particularly advantageous for encoding low-rank matrices. Moreover, complex scattering media, in which the Ising couplings are stochastic variables as in the case of spin-glass systems,^19,20^ are particularly suitable for simulating large combinatorial problems,^21^ while new methodologies are emerging to encode arbitrary problems.^22–24^

All of these groundbreaking approaches suffer from an inherent performance drop that can scale exponentially^25^ with the size of the system. With large systems, the energetic landscape exhibits a spin-glass state,^19^ presenting a multitude of competing energetic minima. As a result, iterative optimization schemes aiming to identify the ground state spin configuration can get stuck in suboptimal solutions. Historically, simulated annealing was one of the first methods relying on a computational virtual “cooling” of the system parameters when combined with Monte Carlo optimization schemes. The cooling process gradually reduces the probability of accepting an iteration that removes the system from the global energetic minimum. The performance of algorithms based on stimulated annealing, however, varies with the choice of problem or complexity of the energetic landscape.^26,27^ The research community has also proposed several classical and quantum adiabatic^28^ protocols – often relying on complex physical architectures - to speed up the convergence time while ensuring a high success rate, including but not limited to holographic evolution,^13^ dimensional annealing,^29^ and simulated adiabatic bifurcation.^30^ As such, solving arbitrarily large and complex Ising problems remains an open challenge in the community. In this work, we propose an Ising machine protocol, i.e., Ising Machine via local and nonlocal Optical Nonlinear (ONIM) detection, and we show that it can enhance the probability of finding the optimal solution to a complex problem with a simple optical implementation.

## Results

### Conceptual Definition

The conceptual scheme is presented in Figure 1. Once the Ising system is defined in terms of spin (solutions) and couplings (problem encoding), its macroscopic energetic state is investigated by sampling a local and a nonlocal nonlinear function of the optical output. Specifically, we refer to the optical field at the detection plane, which can be generally detected in terms of its intensity or field distribution. In our formalism, *H*_*F*_ corresponds to the nonlinear detection of the spatial average of the field. On the contrary, *H*_*I*_ represents the spatial average of the nonlinear local intensity (please refer to SI1 for the full mathematical derivation). First, we explore the energetic landscapes enabled by the two different detection schemes, demonstrating that sampling the energy of a spatially averaged field simplifies the Ising Hamiltonian, removing the emergence of a spin glass state. Finally, we perform energetic annealing of the interference Hamiltonian component, bringing the system from a highly correlated replica state to the ground state of the spin glass landscape of *H*_*I*_, demonstrating an enhanced success rate across parallel simulations.

**Figure 1:**
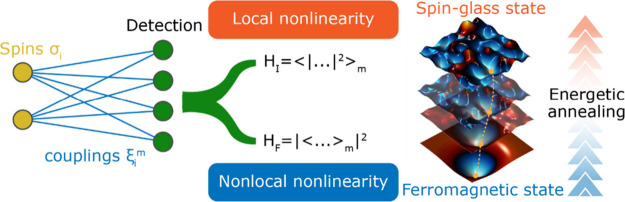
Conceptual scheme for the optical nonlinear Ising machine. The sampling of different nonlinearities – nonlocal *H*_*F*_ and local *H*_*I*_ – shapes the energetic landscape of the Ising problem into a ferromagnetic or spin-glass state, respectively. Energetic annealing is enabled by transitioning between these two nonlinear detections, enhancing the success rate of finding the spin-glass’s ground state.

For simplicity and without loss of generality, Figure 2 presents a proof-of-principle implementation inspired by the recent advances in spatial coherent Ising machines^19,21^ and single-pixel computational imaging.^31–34^ In this scenario, we consider an Ising problem with random couplings implemented through a scattering medium, a classical scenario for testing complex combinatorial problems.^19^ A monochromatic field that is shaped by a phase-only spatial light modulator into binary patterns *E*_*i*_ = *E*(*x*_*i*_,*y*_*i*_) ∈ {−1,1} illuminates a complex media. The probability of light propagating from an initial point *i* to a final point *m* is set by the random complex variable ξ_*i*_^*m*^ which follows circular Gaussian statistics (SI1), leading to a scattered field *E*_*m*_ = ∑_*i=1*_^*N*^ *E*_*i*_ ξ_*i*_^*m*^.

**Figure 2:**
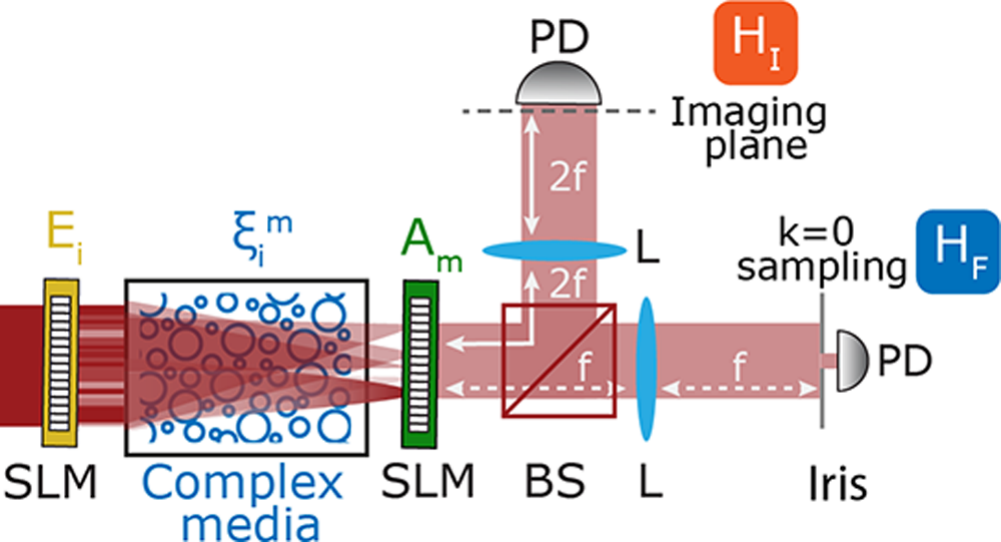
Sketch for a proposed CW spatial optical platform for ONIM using a scattering media. SLM: spatial light modulator; PD: photodetector; L: lens; BS: beam splitter, f: focal length.

Notably, the complex medium provides a natural parallel platform for exploring large-scale complex combinatorial problems, but it can be replaced with appropriate optical shaping techniques, e.g. the complex media transmission matrix could be performed computationally by encoding *E*_*m*_ instead of *E*_*i*_ on the SLM performing the “spins”. The scattered field is further modulated by a spatial function *A*_*m*_ = *A*(*x*_*m*_,*y*_*m*_) ∈ {0,1} before being sampled by two different detection stages measuring the real value of the Hamiltonians *H*_*F,I*_: on one hand, a single-pixel detector placed in the center of the Fourier plane captures the intensity of the spatial average field

1 $$H_{F}=-\overset{N}{\underset{i,j=1}{\sum }}E_{i}E_{j}J_{i,j}^{\left\{m,n\right\}}$$

with

2 $$J_{i,j}^{\left\{m,n\right\}}=-\frac{1}{N}\overset{M}{\underset{m,n=1}{\sum }}\xi_{i}^{m}{\xi^{*}}_{j}^{n}A_{m}A_{n}$$

on the other hand, the second single-element photodetector is placed in the imaging plane with respect to the modulator *A*_*m*_ and measures the spatial average of the local intensity (the full theoretical derivation is presented in SI1)­

3 $$H_{I}=-\overset{N}{\underset{i,j=1}{\sum }}E_{i}E_{j}J_{i,j}^{\left\{m\right\}}$$

with

4 $$J_{i,j}^{\left\{m\right\}}=-\frac{1}{N}\overset{M}{\underset{m=1}{\sum }}\xi_{i}^{m}{\xi^{*}}_{j}^{m}A_{m}^{2}$$

Our scheme is designed to physically implement intensity and field averages using single-point detection. While the main goal of this operation is to carry out a computational task, in the spatial domain this approach avoids the use of optical cameras and carries over some advantages of single-pixel imaging. In particular, our approach is well suited for highly sensitive detection—potentially down to the single-photon level—easing limitations on minimum energy precision measurements.

These two detected Hamiltonians are not independent, as it can be easily verified that *H*_*F*_ = *H*_*I*_ + Δ*H*, where Δ*H* is an interference component arising from the coherent detection performed to obtain *H*_*F*_ (more information in SI5). As shown by eqs 1 and 3, both terms are associated with different Ising Hamiltonians where *E*_*i*_ are the Ising spins and the combinations of ξ_*i*_^*m*^, *A*_*m*_ terms define the spin–spin coupling. Encoding an arbitrary matrix of couplings will then rely on finding the vector *A*_*m*_ that satisfy *J^{m}^*_*i*__,*j*_ = 1/*N*∑_*m*_ ξ_*i*_^*m*^ξ_*j*_^m*^ |*A*_*m*_|^2^, similar to the linear decomposition method where |*A*_*m*_|^2^ are given eigenvalues.^22–24^ To explore the complexity of the energetic landscapes of these two systems, we performed several parallel simulations (or replicas) of a simple Metropolis convergence algorithm, following the Hopfield model^35^ for an Ising system of *N* = 225 spins. Starting from a random initialization for the spins, we flip a single spin and accept the move with probability $e^{\beta \left[I\left(t_{2}\right)-I\left(t_{1}\right)\right]}$ , where *I*(*t*_1,2_) is the intensity calculated at different iteration steps and where $\beta =b\beta_{C}=\frac{b}{T_{c}}$ (*b* ∈ [0,3]) is the inverse of the thermodynamical temperature of the system, which accounts for thermal fluctuations. After 45N iterations, both systems’ evolutions plateau, reaching distinct spin configurations *E*_*k*_ that are compared via the Parisi overlap *q* (SI for details, Figure S1).

In Figure 3, we study the behavior of the system for different values of the parameter α = *M*/*N* (taking values 0.004, 0.02, 0.04, and 1) and using the Hamiltonian *H*_*I*_ and *H*_*F*_ for Figure 3a–d and e–h, respectively. Following the literature, the parameter α, defined as the ratio between the number of output channels and Ising spins, generally accounts for the complexity associated with the resulting Ising system.^19^ The occurrence probability of the Parisi overlap has been estimated at the end of the optimization algorithm for 1200 replicas (SI1).

**Figure 3:**
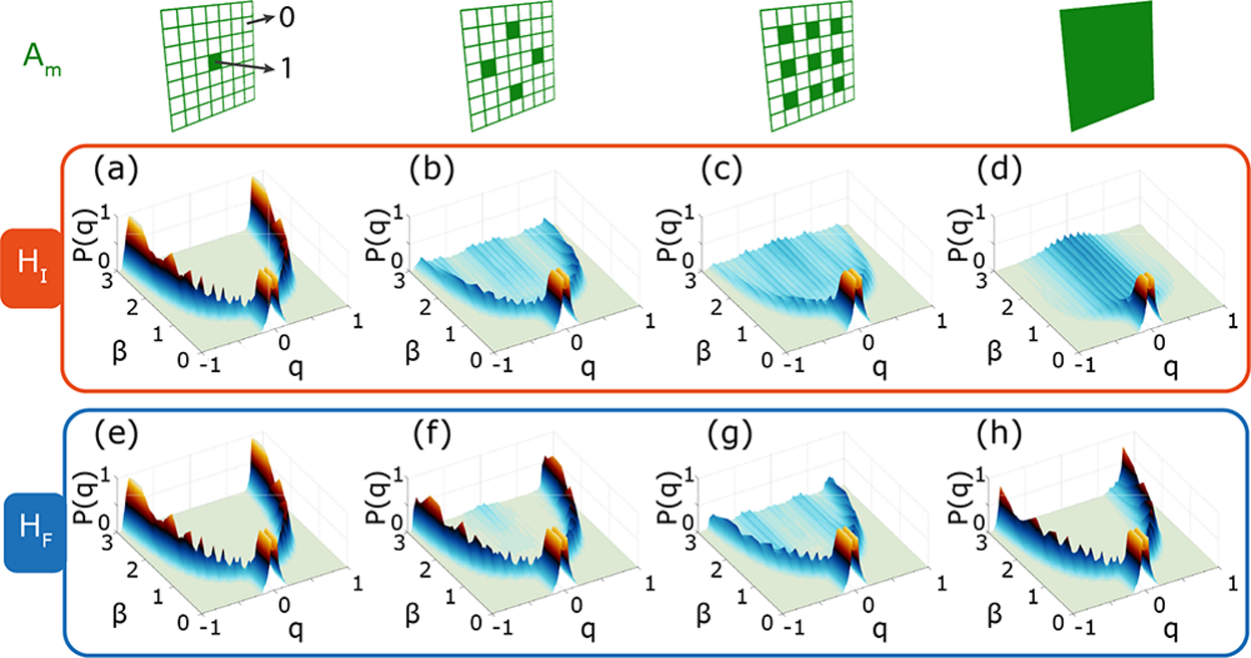
Occurrence probability of the Parisi overlap. (a–d) *H*_*I*_; (e–h) *H*_*F*_. The occurrence probability is evaluated at the end of the optimization algorithm at different values of β = *b*β_*C*_ for (a–d), where *b* ∈ [0,3], and β = *b*β′_*C*_ for (e–h). *M* = 1, 4, 9, *N*. Here, the choice of the spots *M* is random and does not affect the final results.

The Hamiltonian with local nonlinearity *H*_*I*_ (Figure 3a–d) presents a paramagnetic to ferromagnetic transition while decreasing the temperature (increasing β) and for small α. Increasing α → 1, *H*_*I*_ presents a transition from a paramagnetic to a spin-glass state, where the replicas become trapped in multiple local solutions. Conversely, the Hamiltonian with nonlocal nonlinearity *H*_*F*_ (Figure 3e–h) does not exhibit significant changes in behavior for different values of α. On the contrary, the occurrence probability shows a paramagnetic to ferromagnetic transition occurring while increasing β for all cases considered. As a result, *H*_*F*_ always behaves as a single Ising energetic outcome (*m* = 1), whose spins experience couplings with statistical variance proportional to *M* (a full theoretical discussion can be found in SI2). This is due to the fact that the coupling coefficient enabled by the nonlocal nonlinearity detection $J_{i,j}^{\left\{m,n\right\}}=\overset{®}{\xi_{i}\;}\overset{®}{\xi_{j}^{*}\;}$ originates from Gaussian variables $\overset{®}{\xi_{i}\;}=\underset{m}{\sum }\xi_{i}^{m}A_{m}$ , whose variances are *ρ̅* ^2^ = ∑_*m*_ρ^2^ *A*_*m*_ = *M*ρ^2^, and mean values are μ̅ = ∑_*m*_ *μA*_*m*_ = 0 , with *ρ^2^* = 1 and *μ* = 0 the variances and mean values of the *ζ_i_^m^* (SI2), a property that is intrinsically enabled by the detection of Δ*H* interference component (SI3).

The change in the Gaussian variance is directly affecting the critical temperature governing the phase transition from paramagnetic to ferromagnetic state in Figure 3a–d, which for optical glass simulators based on scattering media is expressed as $T_{c}=\rho^{2}\left(1+\sqrt{2\alpha }\right)$ .^19^ Averaging over the disorder before applying the nonlinearity, instead, leads to a new critical temperature for *H*_*F*_ implemented in Figure 3e–h, $T_{c}^{\prime }=M\rho^{2}\left(1+\sqrt{2/N}\right)$ . This leads to an absence of a spin-glass transition for values of α → 1, and at low temperature, all the replica solutions converge to two identical solutions (up to a phase ±1). Such a highly correlated replica state, presented in Figure 3e-h, also holds for small fractions of the interference Hamiltonian component Δ*H* (see Figure S2 in SI1).

In stark contrast to the detection of multiple points of the local nonlinearity *H*_*I*_ accessible via intensity-based cameras,^19^ this result demonstrates that the ability to maximize the transmitted fields through a scattering medium does not depend on the size of the output speckle (or the pinhole used). It is worth noting that the above consideration holds for an arbitrarily chosen probe pattern $A_{m}\in R,A_{m}\in \left[-1,1\right]$ . As such, this suggests that full spatiotemporal field-sensitive waveform control may be extended from a local point^34^ to an arbitrarily chosen delocalized spatial subspace.

Moreover, the duality between the imaging plane and (*k* = 0)-sampling in the Fourier space can be extended to near and far fields. As a result, the same low-level computational complexity of superfocusing light in a spot in the near field of a scattering medium^36^ can be extended to the far field, enabling advanced beam-steering techniques. The critical temperature *T*_*c*_^′^, enabled by far-field or Fourier detection, is also directly related to the measurement of the size *Q* of an opaque object embedded within the scatterer as *T*_*c*_^′^ ∝ *M* = *N* – *Q*.

### Adiabatic Energetic Annealing

The strategic detection of the nonlinearity leads to a tunability of the complexity of the energetic landscape, opening the possibility of emulating quantum-inspired adiabatic computation algorithms to solve optimization problems using a purely classical system. For simplicity and without loss of generality, here we aim to solve an arbitrary complex NP-hard problem, epitomized by the spin-glass state of an Ising Hamiltonian with random couplings *H*_*I*_. In SI4 we report a MAXCUT problem example applied to random edges with *A*_*m*_ = 1.^21^

We perform adiabatic energetic annealing of the interference Hamiltonian component Δ*H*, passing from the nonlocal nonlinear detection *H*_*F*_ to the local *H*_*I*_ following the time-dependent Hamiltonian *H*(*t*) = *H*_*I*_ + γ­(*t*)­Δ*H* (Figures 4 and 5 and SI3, Supporting Information). We perform energetic annealing of the interference component Δ*H* following the Hopfield model (Figure 4): after evolving the system following the Hamiltonian *H*_*I*_ + γ­(*t*)­Δ*H*, we start a linear annealing from γ = 0.0755 at iteration step 5 × 10^3^ and end the annealing (γ = 0) at iteration step 7.5 × 10^4^. We stop the algorithm after 1.5 × 10^5^ steps, where we evaluate the performance. In this set of simulations, the system is composed of *N* = 100 spins, and we consider *M* = 100, β = 7β_*c*_, and 1200 replicas. The thermodynamical temperature is kept constant throughout the annealing and chosen to be in the spin-glass regime for *H*_*I*_. Further analysis of the role of β in the adiabatic energetic annealing are reported in Figures S4 and S5. We notice that, after a transition period between β_*C*_ < β < 6β_*C*_, the performance of adiabatic annealing does not vary substantially, suggesting that for β > 6β_*C*_ the thermal fluctuations are too small to remove the replica from the GS.

**Figure 4:**
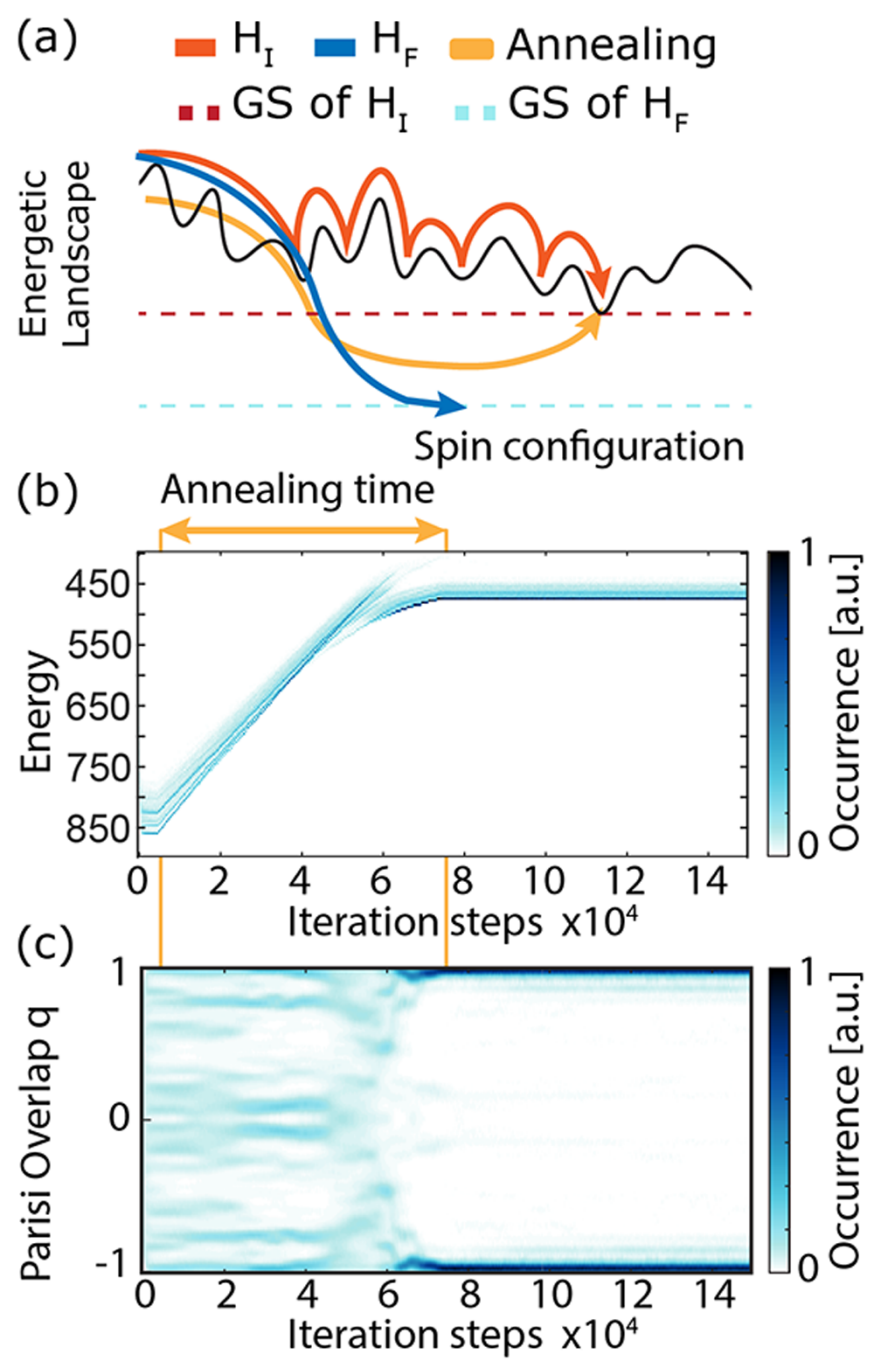
Adiabatic energetic annealing between the nonlocal and local nonlinear detections. (a) Sketch of convergence dynamics on the different Hamiltonians. GS: Ground state. (b) Occurrence of the replicas’ energetic distribution along the iteration time for the annealing Hamiltonian *H*(*t*). (c) Parisi overlap of the replicas undergoing energetic annealing.

**Figure 5:**
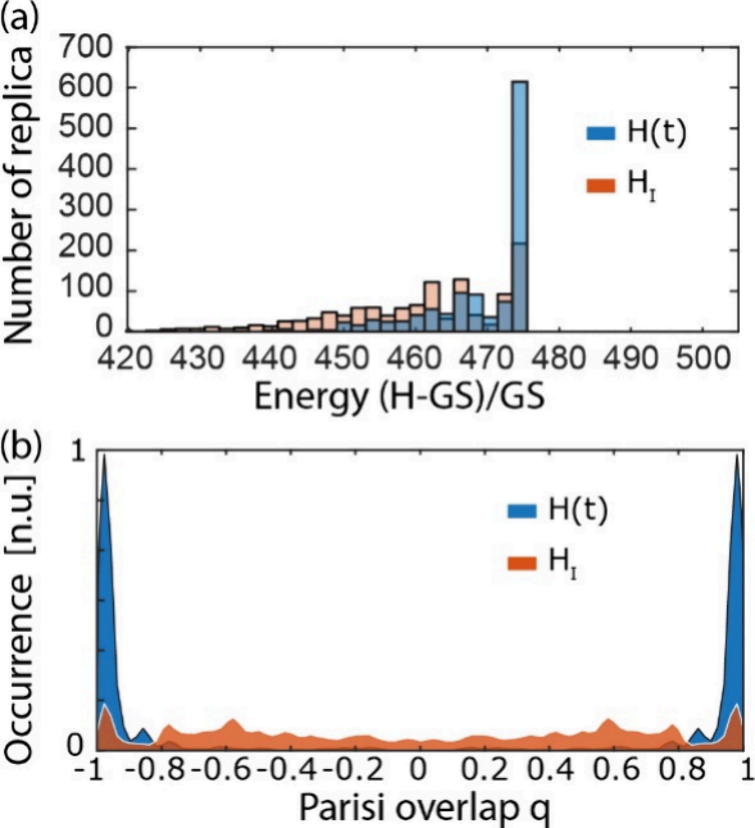
Estimation of success rate. (a) Replica energy distribution compared to the Ground State for *H*_*I*_ (red) and *H*(*t*) (blue), at the end of the energetic annealing process (1.5 × 10^5^ steps). (b) Parisi overlap distribution of the replicas.

As presented in Figure 4c, the system converges initially to a highly correlated replica state, then slowly moves to the ground state of *H*_*I*_ while preserving a high Parisi overlap. This procedure ensures that thanks to the iso-thermal adiabatic transition, most of the replicas converge to the ground state of the spin-glass, with around 50% of the replica in the ground state compared to around 16% of the standard Metropolis algorithm evaluating *H*_*I*_ with 1200 replicas, at low temperature β = 7β_*c*_ and after 1.5 × 10^5^ iterations (Figures 5a, S4, and S5). To verify our final convergence, we computed the energetic value of the ground state GS via simulated annealing optimization routines.^29^ Figure 5 proves that the enhancement of the occurrence of replica solutions in the ground state is not attributable to the Metropolis algorithm alone but instead to the adiabatic evolution presented in Figure 4.

## Discussions and Conclusions

In conclusion, we defined a novel optically based method for tackling large combinatorial problems, namely Optical Nonlinear Ising Machine, inspired by recent field-sensitive manipulation and single-pixel detection technologies.^31–34,37^ Notably, the working principle of ONIM does not rely on a specific physical implementation (e.g., scattering media), and it can be applied to any classical or quantum optical Ising machine architecture based on advanced mixing, e.g. in SPIM-based systems.

The optical nonlinear Ising machine presented in this work shows how, by sampling the local and nonlocal nonlinearity, the dual single-pixel detection reveals the sampling of two deeply connected energetic Hamiltonians: on the one hand, the problem we are trying to solve *H*_*I*_, and on the other hand, an auxiliary “simple” state *H*_*F*_. The energetic difference between the two states Δ*H* corresponds to an interference component that, similarly to quantum fluctuations, is a key element in the simplification of the complexity of the energetic landscape. The ability to tune the complexity of the energetic landscape dynamically, enabled by varying the interference component Δ*H* through tuning the variable γ, enables the implementation of a quantum-inspired adiabatic annealing protocol capable, at a fixed low temperature, of steering most of the replicas toward the ground state of the spin-glass landscape. Compared to conventional parallel isothermal approaches, this translates into a probability enhancement per single replica to find the optimal solution. This represents the first example of classical adiabatic annealing of the interference Hamiltonian component, using a simple optical implementation (i.e., a pair of photodiodes). Further studies will focus on encoding arbitrary couplings in this optical system while comparing the annealing performance.

## Supplementary Material



## Data Availability

The data that support the finding of this study are openly available at https://doi.org/10.17028/rd.lboro.28723271.v1.

## Notes

SI1: Optical nonlinear Ising machine with complex media. Figure S1: Convergence of replicas with different Hamiltonians. SI2: The coupling coefficients *J*_*i*,*j*_^{*m*,*n*}^. SI3: Interference Hamiltonian and adiabatic energetic annealing. Figure S2: Conceptual sketch for the interference energetic component and the adiabatic energetic annealing. Figure S3: Occurrence of the Parisi overlap at different values of the parameter γ. Figure S4: Energetic histogram of the replica states for isothermal adiabatic energetic annealing and Metropolis protocols at different values of β. Figure S5: Occurrence of replica overlap at the final iteration step for isothermal adiabatic energetic annealing and Metropolis protocols at different values of β. SI4: Application to the MAXCUT problem. Figure S6: Application to the MAXCUT problem for all-to-all random couplings (PDF)
