# Supplementary material for: Adiabatic Energetic Annealing via Dual Single-Pixel Detection in an Optical Nonlinear Ising Machine
Source: ACS Photonics. 2025 Apr 14;12(6):2896–901. doi: 10.1021/acsphotonics.4c02496 (PMC12183720; doi:10.1021/acsphotonics.4c02496)
Supplement: Supplementary file 1 [file ph4c02496_si_001.pdf]

Supporting information

Adiabatic Energetic Annealing via Dual Single-Pixel Detection in Optical Nonlinear Ising Machine

Luana Olivieri*, Andrew R. Cooper, Luke Peters, Vittorio Cecconi, Alessia Pasquazi, Marco Peccianti, Juan S. Totero Gongora

Emergent Photonics Research Centre, Dept. of Physics, Loughborough University, LE11 3TU, Loughborough, United Kingdom

* Corresponding author: l.olivieri@lboro.ac.uk

SI1. OPTICAL NONLINEAR ISING MACHINE WITH COMPLEX MEDIA

Without loss of generality, we present the protocol for Optical local and nonlocal Nonlinear detection in an Ising Machine (ONIM), inspired by the recent spatial Ising machines [1,2] and field-sensitive manipulation and single-pixel detection technologies [3–6]. As in spatial Ising machines, we consider an initial modulation of a monochromatic field $E_i = E(x_i, y_i) \in \{-1,1\}$ with N pixels, which illuminates scattering media. Here, E_i represents the Ising spin configuration of an Ising problem. The field in the point i is scattered by the material to a point m with a probability ξ_i^m.

$$\xi_i^m = a_i^m + \imath\, b_i^m \in \mathbb{C}, \tag{S1}$$

where both variables $x = a, b$ are random Gaussian variables distributed as

$$P(x) = 1/\sqrt{2\pi\rho^2}\, e^{-\frac{x^2}{2\rho^2}} \tag{S2}$$

with variance $\rho^2 = 1$ and mean value $\mu = 0$ [7]. The field at the output plane of the scatterer is expressed as

$$E_m = \sum_{i=1}^{N} E_i\ \xi_i^m. \tag{S3}$$

To understand the origin of complexity[7], one can modulate the output plane of the scattering material through a sampling function $A_m = A(x_m, y_m)$, $A_m = 1$ for $m = 1, .., M \leq N$ and $A_m = 0$ elsewhere. Firstly, let's consider the sampling of the local intensity as it would be detected by an optical camera [7,8], or by a bucket photodetector placed in imaging condition with the plane of the modulator A_m.

$$I_l = \sum_{m=1}^{M} |E_m\, A_m|^2 = \sum_{m=1}^{M} \sum_{i,j=1}^{N} E_i\, \xi_i^m\ E_j\, \left(\xi_j^m\right)^*\ A_m^2 \tag{S4}$$

corresponding to the Hamiltonian

$$H_l = -\sum_{i,j=1}^{N} E_i E_j\, J_{i,j}^{\{m\}} \quad ; \quad with\ \ J_{i,j}^{\{m\}} = -\frac{1}{N} \sum_{m=1}^{M} \xi_i^m\, \left(\xi_j^m\right)^*\, A_m^2\ . \tag{S5}$$

Secondly, consider a nonlocal nonlinear sampling – the intensity of the spatial average - as enabled by a single-pixel detection placed in the center of the Fourier space $\left(k_x, k_y = 0\right)$ [3–6]. In this configuration, the spatially-averaged field is expressed as:

$$C = \sum_{m=1}^{M} E_m\, A_m = \sum_{m=1}^{M}\sum_{i=1}^{N} E_i \xi_i^m\, A_m \tag{S6}$$

and the corresponding intensity, expressed as

$$I_F = \left|\sum_{m=1}^{M} E_m\, A_m\right|^2 = \sum_{m,n=1}^{M}\sum_{i,j=1}^{N} E_i\, \xi_i^m\; E_j\, \left(\xi_j^n\right)^*\, A_m A_n, \tag{S7}$$

corresponds to the Hamiltonian

$$H_F = -\sum_{i,j=1}^{N} E_i E_j\, J_{i,j}^{\{m,n\}} \quad ; \quad with \quad J_{i,j}^{\{m,n\}} = -\frac{1}{N}\sum_{m,n=1}^{M} \xi_i^m \left(\xi_j^n\right)^*\, A_m A_n \tag{S8}$$

We explore the energetic landscapes of these Hamiltonians by implementing several parallel Metropolis simulations up to 45N iterations. We first ensure the energetic states plateau at the end of the iteration steps, as presented in Fig. S1.

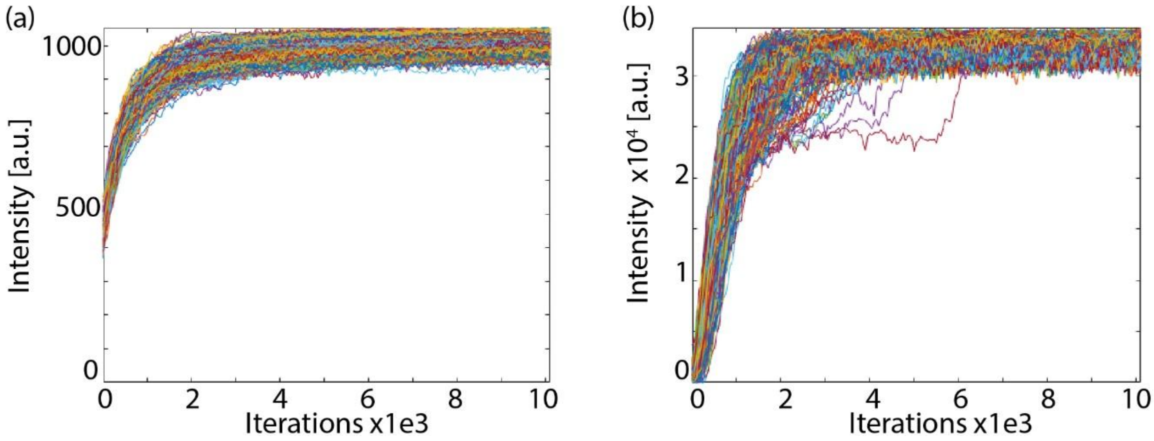

Figure S1: Convergence of replicas with different Hamiltonians. *(a)* H_I, *(b)* H_F .The simulation parameters are *M=225, N=225,* $\beta = 3\beta_c$

We then compare the final spin replica states with indexes α, γ, by calculating the Parisi overlap defined as:

$$q_{\alpha,\gamma} = \frac{1}{N}\sum_{k=1}^{N} E_k^{\alpha} \cdot E_k^{\gamma}, \tag{S9}$$

and specifically addressing their incidence rate (or occurrence probability) $P(q)$.

SI2. THE COUPLING COEFFICIENTS $\mathbf{J_{i,j}^{\{m,n\}}}$

The nonlocal nonlinearity, accessible by sampling the $k = 0$ component of the transmitted field in the Fourier space, gives rise to a Hamiltonian with coupling coefficients

$$J_{i,j}^{\{m,n\}} = -\frac{1}{N}\sum_{m,n=1}^{M} \xi_i^m \left(\xi_j^n\right)^* A_m A_n = -\frac{1}{N}\sum_{m=1}^{M} \xi_i^m A_m \sum_{n=1}^{M} \left(\xi_j^n\right)^* A_n = -\frac{1}{N}\, \overline{\xi_\iota}\, \overline{\xi_j^*} \tag{S10}$$

which do not depend on m, and where

$$\bar{\xi_\iota} = \sum_{m=1}^{M} A_m \xi_i^m \tag{S11}$$

are the averaged values of ξ_i^m over the final states m independently of the value of $A_m \in \mathbb{R},\ A_m \neq \{0\}$. $J_{i,j}^{\{m,n\}}$ is the product of two uncorrelated Gaussian variables with statistic $\bar{\rho}^2 = M\,\rho^2$ and $\bar{\mu} = 0$.

In fact, if we consider two Gaussian independent variables X and Y with variance ρ_X^2, ρ_Y^2, so that $VAR\,(X,Y) = VAR(X) + VAR(Y)$, where $VAR(X) = E(X^2) - E(X)^2$ the sum $Z = aX + bY$ with $a, b \in \mathbb{R}$ is a random variable associated to the variance

$V(Z) = V(aX + bY) = E[(aX + bY)^2] - (E[aX + bY])^2$.

For the sake of completeness:

$E[(aX + bY)^2] = a^2E(X^2) + b^2E(Y^2) + abE(XY)$;

$(E[aX + bY])^2 = \left(aE(X) + bE(Y)\right)^2 = a^2E(X)^2 + b^2E(Y)^2 + abE(X)E(Y)$

$V(Z) = a^2VAR(X) + b^2VAR(Y) + abCOV(X,Y)$

$COV(X,Y) = 0$ for independent Gaussian variables.

As a result, the variance of the Gaussian variables $\bar{\xi_\iota}(\omega_0)$ increases with the number of points M considered:

$$\bar{\rho}^2 = \sum_{m=1}^{M} A_m^2 \rho^2 = M\,\rho^2 \tag{S12}$$

For $A_m = \pm 1$ for $m = 1, .., M$ and $A_m = 0$ elsewhere, and where the ρ^2 is the variance of each independent Gaussian variable, $\rho^2 = 1$ in the simulations.

SI3. INTERFERENCE HAMILTONIAN AND ADIABATIC ENERGETIC ANNEALING

Note that the difference between H_F and H_I gives rise to an energetic "interference" Hamiltonian component

$$\Delta H = H_F - H_I = -\sum_{i=1}^{N} E_i E_j\, J_{i,j}^{\{m\neq n\}}\ ;\ with$$
$$J_{i,j}^{\{m\neq n\}} = -\frac{1}{N} \sum_{m=1, n\neq m}^{M} \xi_i^m \left(\xi_j^n\right)^* A_m A_n \tag{S13}$$

If H_I is interpreted as the joint probability of two photons E_i and E_j reaching the same final point m, then ΔH is the probability that these two photons always reach different states m, n with $m \neq n$ (Fig.S2a). The name "interference Hamiltonian" comes from a loose analogy with calculus applied to complex waves: if $H_F \sim |a + b|^2$, then $H_I \sim |a|^2 + |b|^2$, while $\Delta H \sim ab^* + a^*b$ that maintains the phase "interference" relation of the mixed terms.

We exploit the connection between H_F and H_I to drive the system from the ferromagnetic ground state of H_F to the ground state of H_I following the adiabatic annealing theorem. As reported in Fig.S2b, we perform energetic annealing from the Hamiltonian H_F to H_I

$$H(t) = \gamma(t)H_F + \left(1 - \gamma(t)\right)H_I \tag{S14}$$
$$= H_I + \gamma(t)\Delta H$$

with linear annealing dynamics depending on the iteration step t :

$$\begin{cases} \gamma(t) = \gamma & for\ t \in [0\ 5e3] \\ \gamma(t) : \gamma \to 0 & for\ t \in [5e3\ 7.5e4] \\ \gamma(t) = 0 & for\ t \in [7.5e4\ 1.5e5] \end{cases}$$

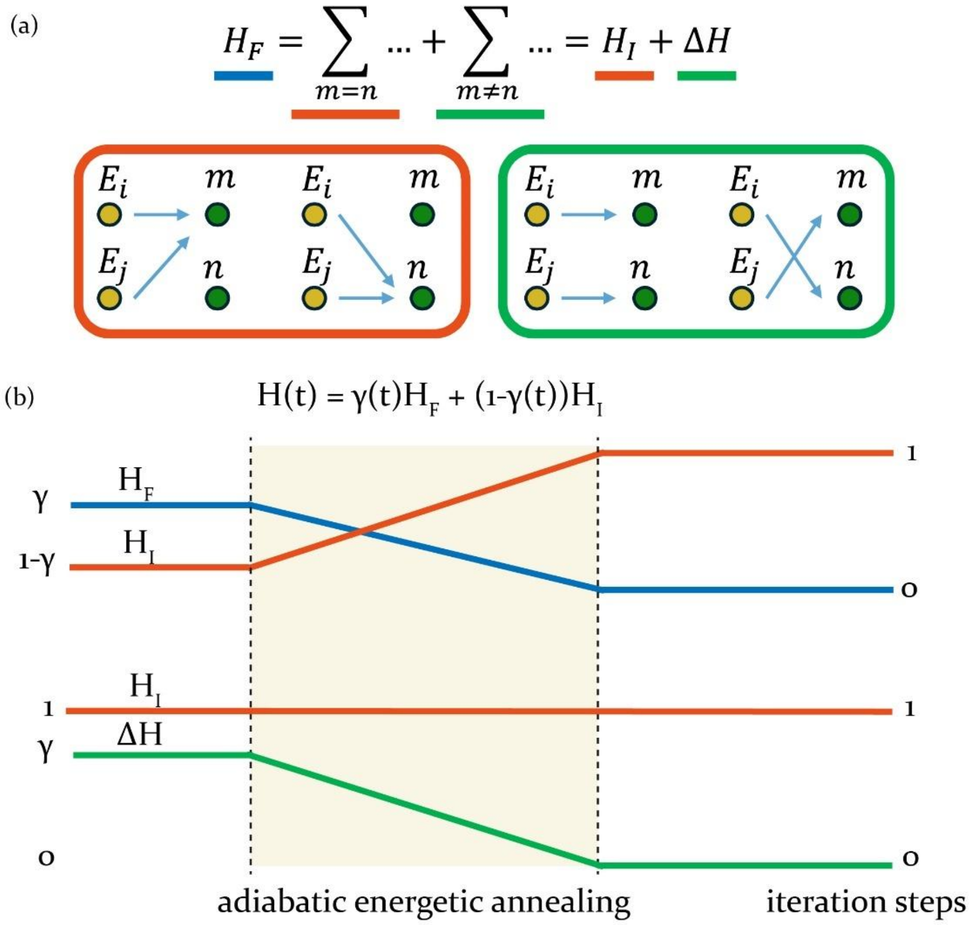

Figure S2: Conceptual sketch for the interference energetic component and the adiabatic energetic annealing. (a) the nonlocal Hamiltonian H_F as a sum of the term H_I and ΔH: H_I is related to the joint probability that two photons always reach the same state m, while ΔH is related to the joint probability of two photons always reaching different states m, n with $m \neq n$. (b) Adiabatic energetic annealing. Slowly switching the energetic optimization goal between the two single-pixel detections H_F and H_I is the same as annealing (i.e., switching off) the Hamiltonian interference component ΔH, while measuring H_I.

To investigate the role of ΔH in correlating the replicas while varying γ, we show in Fig. S3 the replica state after 3×10^3 iteration steps, $\beta = 3\beta_C$, following the Hamiltonian $H_I + \gamma\Delta H$.

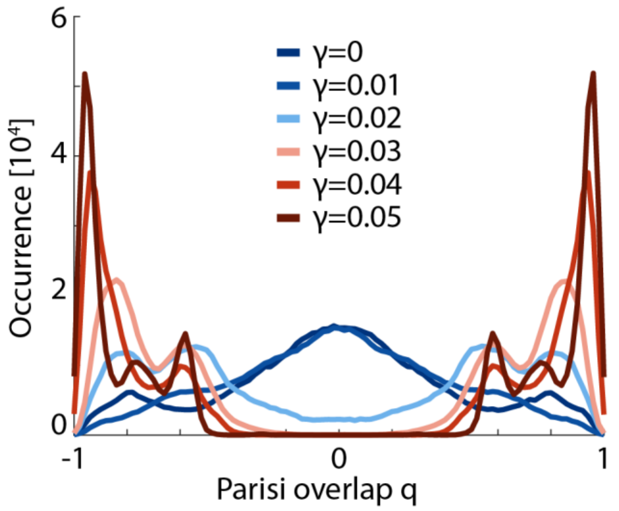

Figure S3: Occurrence of the Parisi overlap at different values of the parameter γ. The simulation parameters are N=100, M=100.

Finally, we present in Fig.S4 the energy histogram of the replicas final state for $H(t)$ (EA) and H_I (Metropolis) and their replica overlap occurrence at different values of $\beta \in [\beta_c, 12\beta_C]$. The results show that a low temperature ($\beta \gg \beta_C$) is required to reduce the effect of thermal fluctuation and reach the GS. The efficiency of the energetic annealing plateaus after $\beta = 6\beta_C$.

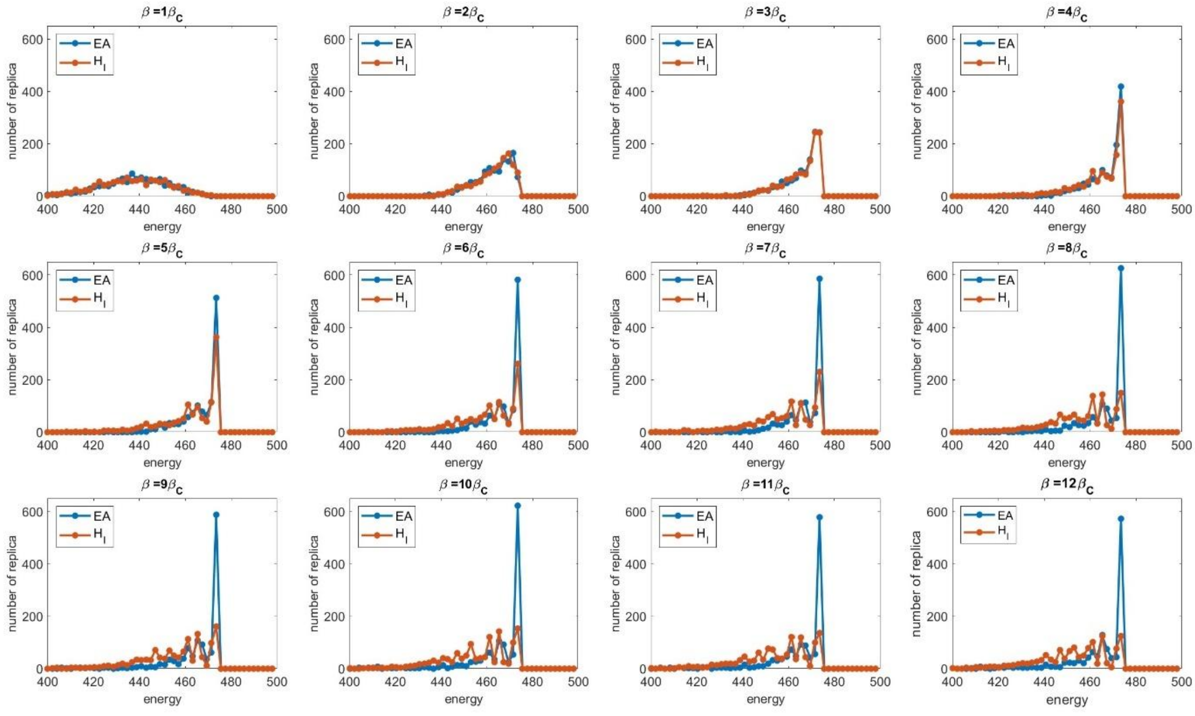

Figure S4: Energetic histogram of the replica states for iso-thermal Adiabatic Energetic annealing and Metropolis protocols at different values of β. The simulation parameters are N=100, M=100, $\gamma = 0.05$, number of replicas 1200, 1.5e5 iteration steps.

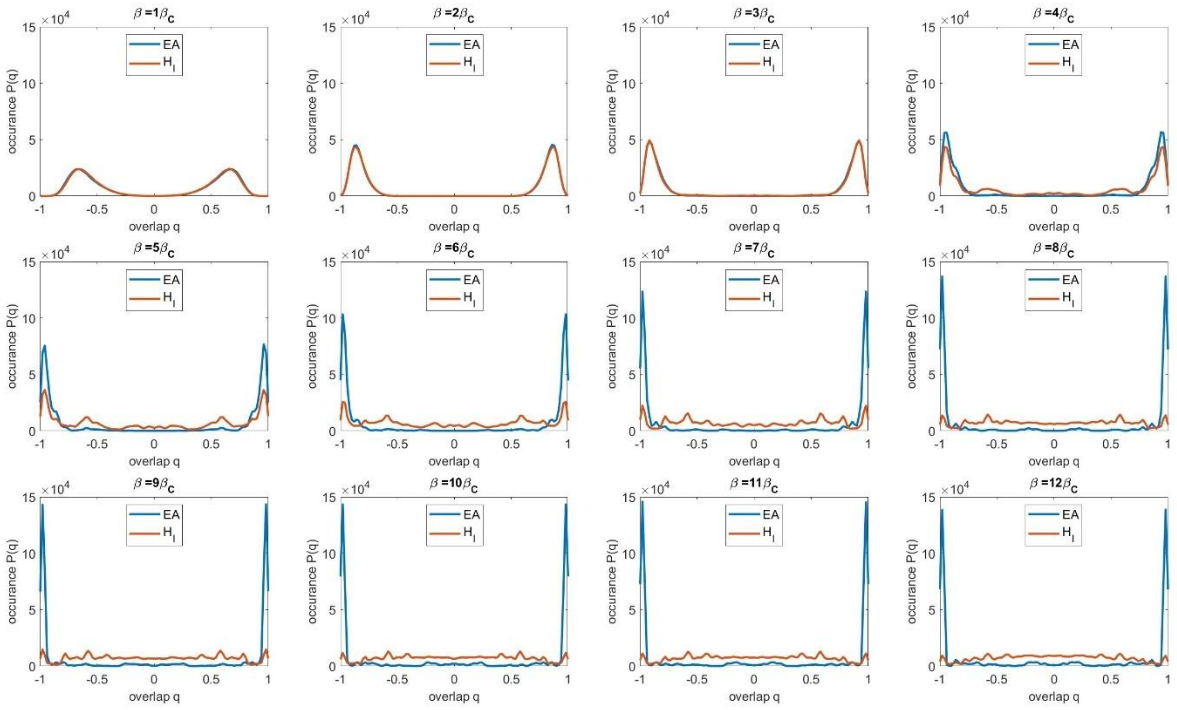

Figure S5: Occurrence of replica overlap at the final iteration step for iso-thermal Adiabatic Energetic annealing and Metropolis protocols at different values of $\boldsymbol{\beta}$. N=100, M=100, $\gamma = 0.05$, number of replicas 1200, 1.5e5 iteration steps.

The ground state has been computed with MATLAB's optimization toolboxes, i.e. using both simulated annealing ***simulannealbnd*** and genetic algorithm protocols ***ga,*** converging to $|GS| = 475.267$.

SI4. APPLICATION TO MAXCUT PROBLEM

In this supplementary section, we present the maximum cut problem (or MAXCUT), an NP-complete task relevant to resource allocation and circuit design[9], and a standard benchmark problem in the literature[10–12]. In particular, Barahona et al. [9] demonstrated that solving a MAXCUT problem is equivalent to finding the ground state of spin glass Hamiltonian of an Ising problem. In other words, if we define a graph G with vertices V and edges $E_{i,j}$ connecting two vertices with weight $c_{i,j} = J_{i,j}$ then

$$H_{ising} + C = \sum_{i,j \in \delta(V)} J_{i,j}$$

With $i,j \in \delta(V)$ are considering only the vertices with opposite signs and $H_{ising} = -\sum_{i,j} J_{i,j}\, \sigma_i \sigma_j$, $\sigma_i = \pm 1$ and $C = \sum_{i,j} J_{i,j}$. So that it becomes clear the task of maximizing the right side of the equation (i.e. MAXCUT) is equivalent to minimizing the Hamiltonian. In the present example, the edge weights are sampled from a random distribution of $J_{i,j}$ in (Fig.S6a). We computed the adiabatic energetic annealing and found a solution that maximise the max cut (Fig.S6b-c). The ground state is populated by 50% of replicas is in agreement with similar studies on CIM[13].

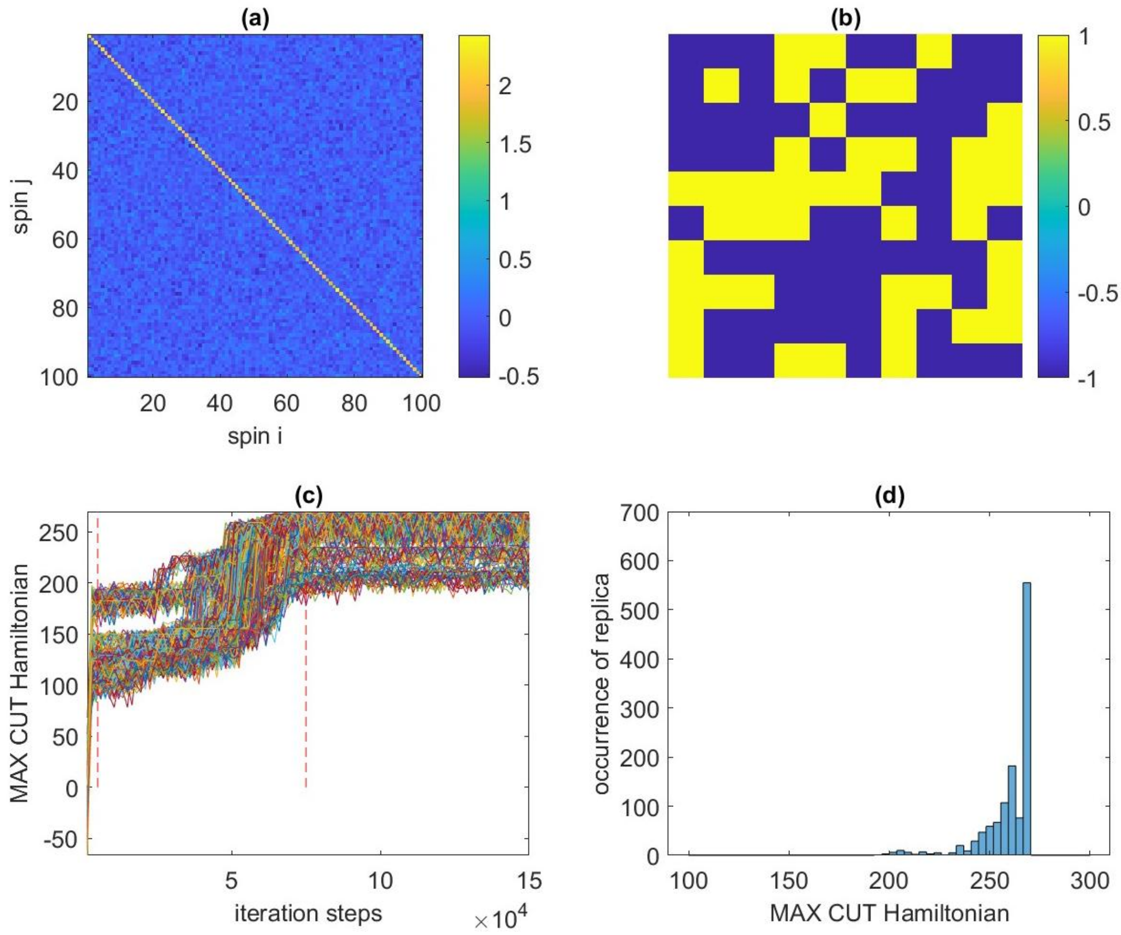

Figure S6: Application to the MAXCUT problem for all-to-all random couplings. (a) Matrix of random couplings $J_{i,j}$; (b) solution found; (c) adiabatic evolution of the MAXCUT Hamiltonian at different iteration steps. Dashed red lines are the start and stop times for the annealing procedure. (d) Histogram of energy for 1200 replicas at the final iteration step.

References

(1) Pierangeli, D.; Marcucci, G.; Conti, C. Large-Scale Photonic Ising Machine by Spatial Light Modulation. *Phys. Rev. Lett.* **2019**, *122* (21), 213902. https://doi.org/10.1103/PhysRevLett.122.213902.

(2) Calvanese Strinati, M.; Pierangeli, D.; Conti, C. All-Optical Scalable Spatial Coherent Ising Machine. *Phys. Rev. Appl.* **2021**, *16* (5), 054022. https://doi.org/10.1103/PhysRevApplied.16.054022.

(3) Olivieri, L.; Gongora, J. S. T.; Peters, L.; Cecconi, V.; Cutrona, A.; Tunesi, J.; Tucker, R.; Pasquazi, A.; Peccianti, M. Hyperspectral Terahertz Microscopy via Nonlinear Ghost Imaging. *Optica* **2020**, *7* (2), 186–191. https://doi.org/10.1364/OPTICA.381035.

(4) Olivieri, L.; Totero Gongora, J. S.; Pasquazi, A.; Peccianti, M. Time-Resolved Nonlinear Ghost Imaging. *ACS Photonics* **2018**, *5* (8), 3379–3388. https://doi.org/10.1021/acsphotonics.8b00653.

(5) Totero Gongora, J. S.; Olivieri, L.; Peters, L.; Tunesi, J.; Cecconi, V.; Cutrona, A.; Tucker, R.; Kumar, V.; Pasquazi, A.; Peccianti, M. Route to Intelligent Imaging Reconstruction via Terahertz Nonlinear Ghost Imaging. *Micromachines* **2020**, *11* (5), 521. https://doi.org/10.3390/mi11050521.

(6) Olivieri, L.; Peters, L.; Cecconi, V.; Cutrona, A.; Rowley, M.; Totero Gongora, J. S.; Pasquazi, A.; Peccianti, M. Terahertz Nonlinear Ghost Imaging via Plane Decomposition: Toward Near-Field

Micro-Volumetry. *ACS Photonics* **2023**, *10* (6), 1726–1734. https://doi.org/10.1021/acsphotonics.2c01727.

(7) Leonetti, M.; Hörmann, E.; Leuzzi, L.; Parisi, G.; Ruocco, G. Optical Computation of a Spin Glass Dynamics with Tunable Complexity. *Proc. Natl. Acad. Sci.* **2021**, *118* (21), e2015207118. https://doi.org/10.1073/pnas.2015207118.

(8) Farhat, N. H.; Psaltis, D.; Prata, A.; Paek, E. Optical Implementation of the Hopfield Model. *Appl. Opt.* **1985**, *24* (10), 1469–1475. https://doi.org/10.1364/AO.24.001469.

(9) Barahona, F.; Grötschel, M.; Jünger, M.; Reinelt, G. An Application of Combinatorial Optimization to Statistical Physics and Circuit Layout Design. *Oper. Res.* **1988**, *36* (3), 493–513. https://doi.org/10.1287/opre.36.3.493.

(10) Pierangeli, D.; Rafayelyan, M.; Conti, C.; Gigan, S. Scalable Spin-Glass Optical Simulator. *Phys. Rev. Appl.* **2021**, *15* (3), 034087. https://doi.org/10.1103/PhysRevApplied.15.034087.

(11) Leonetti, M.; Hörmann, E.; Leuzzi, L.; Parisi, G.; Ruocco, G. Optical Computation of a Spin Glass Dynamics with Tunable Complexity. *Proc. Natl. Acad. Sci.* **2021**, *118* (21), e2015207118. https://doi.org/10.1073/pnas.2015207118.

(12) Gigan, S. Imaging and Computing with Disorder. *Nat. Phys.* **2022**, *18* (9), 980–985. https://doi.org/10.1038/s41567-022-01681-1.

(13) Böhm, F.; Verschaffelt, G.; Van der Sande, G. A Poor Man's Coherent Ising Machine Based on Opto-Electronic Feedback Systems for Solving Optimization Problems. *Nat. Commun.* **2019**, *10* (1), 3538. https://doi.org/10.1038/s41467-019-11484-3.
